# Supplementary material for: Nonlinear noise spectrum measurement using a probability-maintained noise power ratio method
Source: Commun Eng. 2022 Dec 29;1:49. doi: 10.1038/s44172-022-00047-y (PMC10955953; doi:10.1038/s44172-022-00047-y)
Supplement: Supplementary file 2 — Supplementary Information [file 44172_2022_47_MOESM2_ESM.pdf]

Nonlinear noise spectrum measurement using a probability-maintained noise power ratio method: Supplementary Information

Tong Ye[1], Xiaofei Su[1], Ke Zhang[1], Chengwu Yang[1], Jingnan Li[1], Yangyang Fan[1], Hisao Nakashima[2], Takeshi Hoshida[2], Zhenning Tao[1]*

[1] Fujitsu Research and Development Center, No.8 Jianguomenwai Ave, Chaoyang District, Beijing, China

[2] Fujitsu Limited., 1-1 Kamikodanaka 4-Chome, Nakahara-ku, Kawasaki 211-8588, Japan

taozn@fujitsu.com

Supplementary Discussion

At first, the application range of Probability-Maintained (PM) Noise Power Ratio (NPR) method is discussed. The equivalent additive noise model approximates the nonlinear distortion as an additive noise having specific spectrum. Since nonlinear system is a broad concept, we would not say the PM NPR method could be applied to all nonlinear systems. However, the verified successful cases include the general Volterra model, the vertical cavity surface-emitting laser (VCSEL) and distributed feedback (DFB) laser cases dominated by the 2nd-order nonlinearity, the case of the electrical driver dominated by the 3rd-order memory nonlinearity, the optical coherent transmitter case having sine nonlinear function, the electrical DAC case having 3rd-, 5th-, 7th-, and 9th-order nonlinearities, the case of fiber nonlinear Kerr effect in both single span and multi-span transmissions. In addition, many nonlinear impairment sources in real-world communication applications including electrical circuits, the electrical digital-to-analog converter (DAC), optical devices (VCSEL, DFB laser), the optical coherent transmitter, and the optical fiber are verified. The proposed method is quite universal. The searching for exceptional cases is left for future research.

Secondly, the complexity and implementation considerations are discussed. Proposed PM NPR method is quite simple to be used compared with conventional NPR method and orthogonal decomposition method. The digital signal processing of PM notch is more complex than that of the simple notch and orthogonal decomposition. However, such a process is performed only once in the design stage of the test symbol sequence. The test symbol sequence obtained by one person could be used by any other person at any time. Having the PM notch symbol sequence, the only thing is to replace the actual transmitted symbol sequence by the PM notch symbol sequence. Then the spectrum at output port of the system under test is measured. Conventional NPR method needs special instrument or band-stop device to implement the notch process.

The orthogonal decomposition method is easy to understand, the digital signal processing is not difficult, and it could obtain the entire equivalent noise spectrum. Furthermore, the system performance estimation based on orthogonal decomposition is accurate. It looks very nice. However, the real implementation of orthogonal decomposition is prohibitively challenging and tricky because it needs the accurate measurement and comparison of the input signal and the output signal[1]. The reasons are illustrated in following.

a) The orthogonal decomposition method needs very expensive measurement instruments, such as the high-speed arbitrary waveform generator and the digital storage oscilloscope. The price of such instrument is several times higher than the spectrum analyzer used in the PM notch method.
b) The orthogonal component $y_o(t) = y(t) - y_c(t)$ is a small difference between two large signals. A small measurement error of a large signal caused a significant error in the orthogonal component. Supposing the NPR is -20 dB, then the power of the $y(t)$ will be 100 times larger than that of the $y_o(t)$. If the measurement error of the $y(t)$ is 1%, the error of the $y_o(t)$ is comparable with the $y_o(t)$ itself.
c) Some nonlinear device has different types of input and output signals. For example, in the case of high-bandwidth coherent driver modulator (HB-CDM) which is the main nonlinear device in the optical coherent transmitter, the input signal is an electrical analog signal, whereas the output signal is an optical signal.

Comparison of electrical signal and optical signal is very challenging and tricky[1].

d) For the cases of fiber Kerr effect, there are the laser phase noise, the amplifier spontaneous emission noise, and the polarization uncertainty in the real-world transmission. Those unknown noises also cause large errors in orthogonal decomposition.

The proposed PM NPR method only needs the transmitter to send a predefined sequence and a spectrum analyzer to measure the output spectrum. All above difficulties are overcome.

Thirdly, the noise probability distribution function (PDF) selection is discussed. Nonlinear distortion is a very complicated phenomenon. "Finding the accurate PDF of the nonlinear distortion" is a possible way to estimate the nonlinear system performance. However, the one-dimensional PDF is not sufficient. The multi-dimensional PDF of nonlinear distortions at different times should be used. Considering that the receiver has a linear equalizer, the noise after the equalizer is the linear combination of nonlinear distortions at different times before the equalizer. Since the PDF of nonlinear distortion is not Gaussian, only the spectrum information is not sufficient. Achieving the multi-dimensional PDF is very difficult.

The object of our approach is to estimate the nonlinear system performance accurately and practically but not to reproduce the complex nonlinear distortion. Consequently, our solution is to use the X-distributed noise passing through the noise spectrum profile filter to approximate the very complex nonlinear distortion so that the nonlinear system performance could be estimated practically. For the medium-level additive white Gaussian noise (AWGN) case, the choice of noise distribution does not make much difference, and the power spectrum of additive noise is sufficient to describe system performance. For the case with nonlinear noise significantly larger than AWGN, the distribution of equivalent additive noise should be considered carefully. Gaussian noise is a more natural choice for noise distribution, which is an implicit assumption in calculating signal-to noise ratio[2] (SNR), error vector magnitude[3,4], and so on. However, both simulations and experiments show that the selection of zero-mean Chi-square noise has better performance in the case of large memory and low-order nonlinearity. In the case of DAC nonlinearity, the selection of Gaussian noise has better performance because there are multiple nonlinear terms with different orders. We have shown the physical explanation of the noise selection for different cases including the long memory and low order nonlinearity, the DAC nonlinearity, the distributed nonlinearity of the multi-span optical fiber transmission. Since it is not a strict mathematical proof, there may be other noise selections that outperforms Gaussian and zero-mean Chi-square in some nonlinear cases. It is left for future research.

Supplementary Methods

Supplementary Fig. 1 PDFs of different signals in PM notch iteration

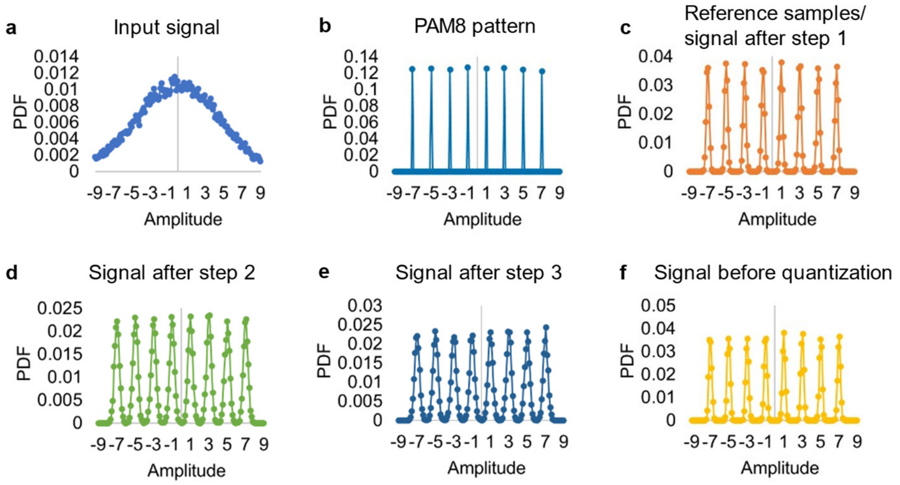

Probability distribution functions (PDF) of the input random signal (**a**), PAM8 pattern (**b**), reference

samples after diffusion and the signal after step 1 (**c**), signal after step 2 (**d**), signal after step 3 (**e**), and diffused signal before 3-bit quantization (**f**).

In the supplementary methods, we will explain the generation of PM notch symbol sequence (Fig. 2 in the article) in detail. Fig. 2ai shows the flow diagram. It consists of three steps in each iteration and a final quantization after the iteration.

Before the iteration, the reference samples are generated firstly. Here we take a PAM8 pattern (Supplementary Fig. 1b) with a length of 128k as an example. The reference samples (Supplementary Fig. 1c) are the random PAM8 pattern plus a diffusion which is a 128k-length white Gaussian noise. This Gaussian noise has a zero mean, and its power is -30 dB of the PAM8 pattern. The initial input signal (Supplementary Fig. 1a) could be any kind of random signal, such as a white Gaussian random signal.

The first step is "Construct PDF". In this step, the samples of the input signal (initial or iterated input signal) are replaced by the reference samples. An example for this replacement is shown in Fig. 2aii of the article. The amplitudes of reference samples and input signal are sorted, and their orders are recorded. Then the samples of input signal are replaced by reference samples according to the recorded order. For example, if the maximum sample of the input signal is found at time index 19, it is replaced by the maximum sample in the reference samples. After replacing, the new signal sequence has exactly the same PDF of the reference samples (Supplementary Fig. 1d). This is the meaning of "Construct PDF". Of cause, such replacement changes the power spectrum density (PSD) of the signal sequence. However, the PSD change is not significant because the order is kept in the replacement.

The 2nd step is the "Adjust spectrum", as shown in Fig. 2aiii of the article. The spectrum of generated signal sequence after step 1 (Construct PDF) is divided into many resolution blocks. The "resolution block" has the same concept as the resolution bandwidth of a spectrum. For example, if the length of sequence is 128k and its baud rate is 21 Gbaud. The total signal bandwidth (−10.5 GHz ~ 10.5 GHz) is divided into 8192 resolution blocks. The resolution bandwidth is 2.56 MHz (21GHz/8192), and each block has 16 (128k/8192) frequency points. Step 2 has two parts: one is a random perturbation within the resolution block, and the other is adjusting the total power of each resolution block equal to the power of corresponding block in the spectrum of reference samples. The "perturbation" means the frequency components (16 frequency points) within the 2.56 MHz resolution block is multiplied by a random value 1+p*N (0,1). A typical value of p is 0.05 and N (0,1) is a standard-Gaussian sequence with a length of 16. In each iteration, the perturbation is randomly chosen. We will illustrate the necessity of this random perturbation after introducing step 3.

The 3rd step is notching the signal spectrum. Notching one or more frequency slots are both permitted. Here the "notching" means setting the PSD of frequency points within notch bandwidth to zero. In this example, the notch bandwidth is 954.5 MHz. The notching process keeps a deep notch in the spectrum, but it changes the PDF (Supplementary Fig. 1e). Then, the difference between the PDF of the generated signal sequence and that of reference samples are calculated as $PDF\ difference = \frac{1}{2}\sum_N \left|PDF_{gen}(i) - PDF_{ref}(i)\right|$. N is the total number of bins in calculating the PDFs, and i is the bin index. If the PDF difference is smaller than the threshold, the desired signal sequence which has a notch in the spectrum and has the same PDF as reference samples is found and the iteration stops (Supplementary Fig. 1f). If not, return to step1. Finally, the signal sequence is quantized to PAM8. It's worthy to notice that the process of PM-notch is nothing but to find a special PAM8 symbol sequence which has a notch in the spectrum and has the same PDF as the random PAM8. It is carried out only ONCE in the design stage of the test signal. The test symbol sequence obtained by one person could be used by any other persons at any time. Thus, the complexity of PM notch process is not an issue.

Supplementary Fig. 2 PDF difference between the generated signal and reference samples.

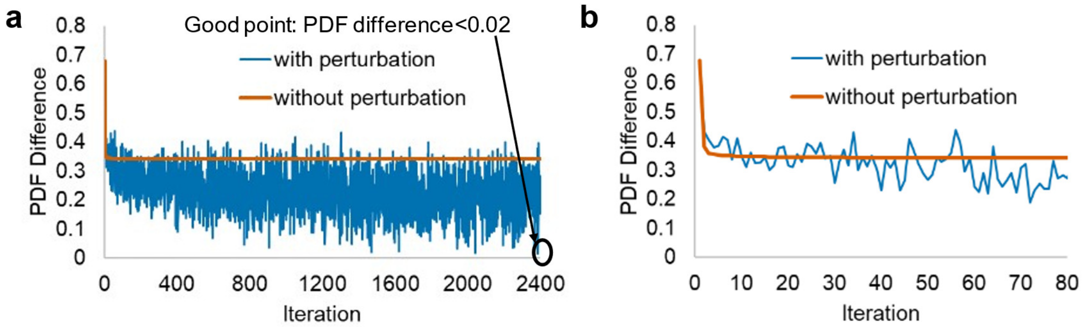

a Results from the 1 iteration to 2400 iterations **b** Detailed results from 1 to 80 iterations. The random perturbation helps to find a good point with small PDF difference.

The necessity of “perturbation” is illustrated in following. Supplementary Fig. 2 shows the PDF difference changes with iteration index when the perturbation is and is not used. If the perturbation is not used, the PDF difference decreases at first and saturates at about 0.34 quickly. The sequences after saturation are exactly same and the PDF difference has 0.34. The iteration falls into the local optimum point.

The perturbation process keeps the overall spectrum beyond the resolution block but changes the details within the resolution block. From the time domain point of view, “the change within the resolution block” means that the signal sequence changes. In other words, “perturbation” generates another signal sequence but keeps the overall spectrum same. Many different signal sequences are hit during the iteration because the perturbation is random. The fluctuation of PDF difference in Supplementary Fig. 2a shows the this. Among those signal sequences, we could select a good one with enough small PDF difference and stop the iteration. For example, the point in Supplementary Fig. 2a has a PDF difference lower than 0.02. The iteration is a “random searching” process and “convergence” is not necessary.

Supplementary References

1. Tao, Z. et al. Characterization, Measurement and Specification of Device Imperfections in Optical Coherent Transceivers. *J. Lightwave Technol.* **40**, 3163-3172 (2022).
2. Pedro, J. C., Carvalho, N. B. & Lavrador, P. M. Modeling nonlinear behavior of band-pass memoryless and dynamic systems. *Proc IEEE MTT-S International Microwave Symposium Digest*, 2133-2136 Vol.3 (2003).
3. Gharaibeh, K. M., Gard, K. G. & Steer, M. B. Accurate estimation of digital communication system metrics - SNR, EVM and /spl rho/ in a nonlinear amplifier environment. *Proc 64th ARFTG Microwave Measurements Conference, Fall 2004.*, 41-44 (2004).
4. Banelli, P. & Cacopardi, S. Theoretical analysis and performance of OFDM signals in nonlinear AWGN channels. *IEEE T. Commun.* **48**, 430-441 (2000).
