# Supplementary material for: Nonlinear noise spectrum measurement using a probability-maintained noise power ratio method
Source: Commun Eng. 2022 Dec 29;1:49. doi: 10.1038/s44172-022-00047-y (PMC10955953; doi:10.1038/s44172-022-00047-y)
Supplement: Supplementary file 3 — Description of Additional Supplementary Files [file 44172_2022_47_MOESM3_ESM.pdf]

Description of Additional Supplementary Files

File name: Supplementary Data 1-1

Description: Row data of Figure 1

File name: Supplementary Data 1-2

Description: Row data of Figure 2

File name: Supplementary Data 1-3

Description: Row data of Figure 3

File name: Supplementary Data 1-4

Description: Row data of Figure 4

File name: Supplementary Data 1-5

Description: Row data of Figure 5

File name: Supplementary Data 1-6

Description: Row data of Figure 6

File name: Supplementary Data 1-7

Description: Row data of Figure 7

File name: Supplementary Data 1-8

Description: Row data of Figure 8

File name: Supplementary Data 1-9

Description: Row data of Figure 9

File name: Supplementary Data 1-10

Description: Row data of Figure 10

File name: Supplementary Data 2

Description: Coefficients of 3rd-order Volterra model.

File name: Supplementary Data 3

Description: This supplementary file lists the generated PM notch signals with 6 different notch frequencies.

File name: Supplementary Data 4

Description: Row data of Supplementary figures
